# Co-culturing nucleus pulposus mesenchymal stem cells with notochordal cell-rich nucleus pulposus explants attenuates tumor necrosis factor-α-induced senescence

**DOI:** 10.1186/s13287-018-0919-9

**Published:** 2018-06-26

**Authors:** Xiao-Chuan Li, Mao-Sheng Wang, Wei Liu, Cheng-Fan Zhong, Gui-Bin Deng, Shao-Jian Luo, Chun-Ming Huang

**Affiliations:** Department of Orthopaedic Surgery, Gaozhou People’s Hospital, Guangdong, 525200 China

**Keywords:** Intervertebral disc degeneration, Notochordal cell-rich nucleus pulposus explants, Nucleus pulposus mesenchymal stem cells, Senescence, TNF-α

## Abstract

**Background:**

Cell therapy for the treatment of intervertebral disc degeneration (IDD) faces serious barriers since tissue-specific adult cells such as nucleus pulposus cells (NPCs) have limited proliferative ability and poor regenerative potential; in addition, it is difficult for exogenous adult stem cells to survive the harsh environment of the degenerated intervertebral disc. Endogenous repair by nucleus pulposus mesenchymal stem cells (NPMSCs) has recently shown promising regenerative potential for the treatment of IDD. Notochordal cells (NCs) and NC-conditioned medium (NCCM) have been proven to possess regenerative ability for the treatment of IDD, but this approach is limited by the isolation and passaging of NCs. Our previous study demonstrated that modified notochordal cell-rich nucleus pulposus (NC-rich NP) has potential for the repair of IDD. However, whether this can protect NPMSCs during IDD has not been evaluated.

**Methods:**

In the current study, tumor necrosis factor (TNF)-α was used to mimic the inflammatory environment of IDD. Human NPMSCs were cocultured with NC-rich NP explants from healthy rabbit lumbar spine with or without TNF-α. Cell proliferation and senescence were analyzed to investigate the effect of NC-rich NP explants on TNF-α-treated NPMSCs. The expression of mRNA encoding proteins related to matrix macromolecules (such as aggrecan, Sox-9, collagen Iα, and collagen IIα), markers related to the nucleus pulposus cell phenotype (including CA12, FOXF1, PAX1, and HIF-1α), and senescence markers (such as p16, p21, and p53), senescence-associated proinflammatory cytokines (IL-6), and extracellular proteases (MMP-13, ADAMTS-5) was assessed. The protein expression of CA12 and collagen II was also evaluated.

**Results:**

After a 7-day treatment, the NC-rich NP explant was found to enhance cell proliferation, decrease cellular senescence, promote glycosaminoglycan (GAG), collagen II, and CA12 production, upregulate the expression of extracellular matrix (ECM)-related genes (collagen I, collagen II, SOX9, and ACAN), and enhance the expression of nucleus pulposus cell (NPC) markers (HIF-1α, FOXF1, PAX1, and CA12).

**Conclusion:**

Modified NC-rich NP explants can attenuate TNF-α-induced degeneration and senescence of NPMSCs in vitro. Our findings provide new insights into the therapeutic potential of NC-rich NP for the treatment of IDD.

## Background

Intervertebral disc degeneration (IDD) is the pathological basis of lower back pain, and current treatment options are mainly aimed at relieving pain symptoms; however, these fail to repair the degenerated intervertebral disc (IVD) [1]. The main characteristics of age-related IDD are a decrease in resident cells and a decline in extracellular matrix (ECM) deposition [2]. Therefore, it has been suggested that resident cells play an important role in maintaining nucleus pulposus (NP) homeostasis by sustaining cell numbers and synthesizing new matrix [3]. During IDD, the number of resident cells decreases; therefore, restoring the degenerating disc using healthy cells would represent a logical treatment strategy.

Over the past decades, several reports have proven that mesenchymal stem cells (MSCs) can differentiate into nucleus pulposus-like cells [4, 5]. Meanwhile, nucleus pulposus cells (NPCs), used as tissue-specific adult cells, were reported by many studies for treating IDD. However, NPCs have limited proliferative ability and poor regenerative potential; moreover, exogenous adult stem cells cannot survive in the degenerated IVD environment due to the associated harsh conditions such as strong forces, acidic pH, hypoxia, hyperosmolarity, and limited nutrients [6]. Hence, studying endogenous repair might provide us with a new method for regenerative therapy to treat IDD.

Endogenous tissue repair, which relies on the regenerative ability of tissue-specific stem cells (SCs), has been verified in human liver, skin, muscle, nervous system, heart, and bone [7, 8]. To date, studies on the regenerative potential of endogenous repair mechanisms have only begun to shed light on IDD in many animal models and in humans. Risbud et al. were the first to demonstrate the existence of progenitor cells in the NP [9]; subsequently, several studies have proposed the existence of SCs in mouse, rabbit, porcine, canine, bovine, and rhesus monkey NP tissue [10–13]. Moreover, nucleus pulposus mesenchymal stem cells (NPMSCs) have shown promising regenerative potential for IDD [14]. Unfortunately, several recent studies have reported decreases in cell numbers and the deterioration of NPMSC properties during IDD [15, 16]. Based on this, a promising strategy would stimulate native NPMSC proliferation and differentiation during this condition.

An increasing number of studies has demonstrated that disc degeneration is mainly associated with inflammation [17]. Inflammatory cytokines can significantly promote cell degeneration, whereas cell degeneration is also related to increases in inflammatory cytokines [18]. Tumor necrosis factor (TNF)-α, a typical inflammatory cytokine, can increase cell senescence, autophagy, apoptosis, and proliferation [19]. Many in-vitro studies have reported that TNF-α triggers a range of pathogenic responses in the disk cells and also affects disc vitality in vivo via transfer through the endplate [20]. This suggests that TNF-α plays a critical role in IDD. It is important to understand the process of NP degeneration to uncover an effective strategy to repair the damage associated with this condition. Based on these facts, we deduced that inhibiting inflammatory cytokine-induced disc degeneration might be a possible strategy for the prevention and treatment of this condition.

To date, notochordal cells (NCs) have been found in the embryonic notochord structure of chordates [21]; human NCs disappear at approximately age 10, whereas the degeneration of the IVD is initiated shortly after this [22]. Subsequently, NCs were considered to hold potential for the regeneration of IDD by improving NPC viability and the expression of matrix proteins [23–25]. Several recent studies have suggested the robust regenerative potential of NCs or notochordal cell-conditioned medium (NCCM) for the treatment of IDD, and that this probably occurs through the stimulation of resident NPCs via secretory factors [23, 26, 27]. However, it is very difficult to promote the proliferation of NCs in vitro because these cells are sensitive to nutrient deprivation in this environment. In addition, NCCM can lose some trophic factors during production and storage processes. Hence, novel modifications based on the regenerative potential of NCs are urgently needed to deal with these shortages. Our previous research demonstrated that modified NC-rich NP explants can improve cell proliferation, cell viability, and NC phenotype [28]. However, whether this improvement can maintain the regenerative function of NCs has not been investigated. In this study, the biological potential of NC-rich NP in the protection of TNF-α-treated NPMSCs was tested. Moreover, endogenous repair of IDD might provide an additional means to obtain sufficient ECM and cells to achieve a healthy NP. Therefore, the aim of this study was to investigate the effects of NC-rich NP explant coculture on cell morphological features, proliferation, senescence, and extracellular gene/protein expression in NPMSCs.

## Methods

### Ethics statement

Human NP tissue was harvested from four male patients with a diagnosis of lumbar disc herniation and where the degeneration degree was Pfirrmann grade II. The age range was 37–52 years, with a mean age of 45.5 years. Informed consent was obtained from each patient. In addition, 36 3-month-old male New Zealand white rabbits (weight 1500–2000 g) were used in this study. The experiment was performed according to the amended declaration of Helsinki and was approved by the Committee of Gaozhou People’s Hospital (no. 2016–007).

### Harvesting rabbit NCs, the NC-rich NP explant model, and sample grouping of samples

NC-rich NP was harvested according to our previous procedure [28]. Briefly, after intravenous general anesthesia (3% pentobarbital sodium, 1 ml/kg), the rabbits were sacrificed and the NC-rich NP tissue from L4/5, L5/6, or L6/7 was harvested and washed with Dulbecco’s modified Eagle’s medium (DMEM) containing 100 U/ml penicillin/streptomycin. Subsequently, NP tissue was partially digested with 0.1% collagenase II (Sigma-Aldrich) for 2 h. Then, all samples were placed in 24-well transwell inserts in triplicate and incubated in culture medium at 37 °C under 5% CO_2_. The medium was removed and replaced every second day.

**Fig. 1:**
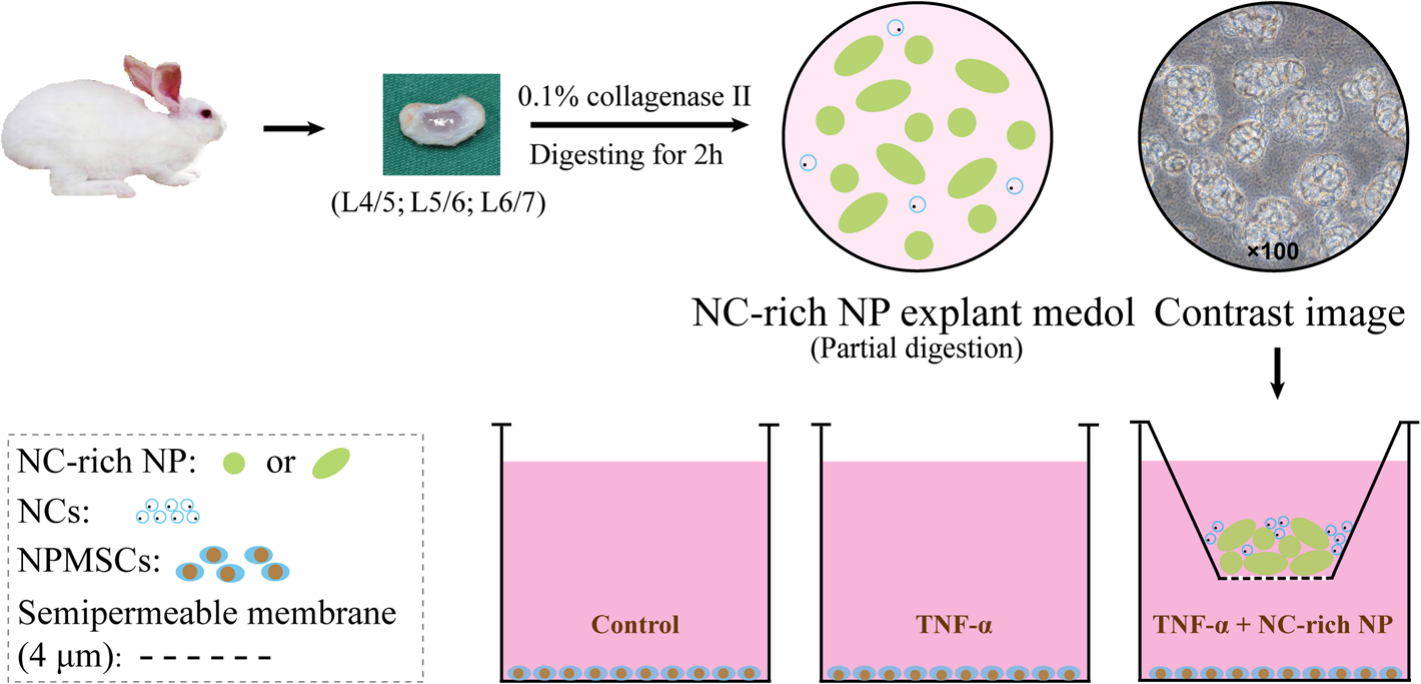
Process of harvesting notochordal cell (NC)-rich nucleus pulposus (NP) explants model and grouping. Rabbit NP tissue from L4/5, L5/6, or L6/7 IVDs were harvested and partially digested to harvest NC-rich NP for the explant model. Human nucleus pulposus mesenchymal stem cells (NPMSCs), incubated in 24-well transwell inserts, were cultured in serum-free medium for 24 h. Subsequently, samples were divided into three groups in triplicate as follows: group 1 was treated as the control, group 2 was treated with 10 ng/ml tumor necrosis factor (TNF)-α, and group 3 was treated with 10 ng/ml TNF-α and NC-rich NP as the coculture group. After 7 days of culture, NC-rich NP explants and NPMSCs were harvested for further analysis

NCs were isolated from rabbit NP tissue and digested with 0.2% collagenase II (Sigma-Aldrich) for 6 h. Cells were then cultured in culture medium at 37 °C under 5% CO_2_. To determine whether NC-rich NP explants can inhibit TNF-α-induced NPMSC senescence, cells were cultured in low-serum medium (DMEM/F-12 supplemented with 1% fetal bovine serum (FBS)). A diagram of the schematic is shown in Fig. 1. After culture in serum-free medium for 24 h, samples were divided into three groups as follows: group 1 was treated with NC-rich-NP as the control, group 2 was treated with 10 ng/ml TNF-α, and group 3 was treated with 10 ng/ml TNF-α and NC-rich NP explant as the coculture group.

### Isolation of human NPMSCs

NPMSCs were isolated and harvested as previously reported [28]. Briefly, NP samples were collected and immediately transported to a cell culture room under sterile conditions. After the annulus fibrosus and cartilaginous endplates were removed and washed at least three times, the NP tissues were mechanically minced into small pieces (< 1 mm^3^) and digested with 0.2% collagenase II (Sigma, USA) for 4 h at 37 °C in a humidified incubator. The suspended cells were then filtered with a 200-μm mesh filter and centrifuged at 200 × *g* for 5 min, which was followed by two washes with phosphate-buffered saline (PBS). Finally, the cell pellets were cultured as an explant in standard MSC expansion medium, consisting of low-glucose DMEM (HyClone), 10% fetal calf serum (Gibco), and 1% penicillin/streptomycin (Gibco) in 25-cm^2^ cell culture flasks at a density of 1 × 10^5^ cells/ml; cells were cultured in a humidified incubator at 37 °C under 5% CO_2_.

**Fig. 2:**
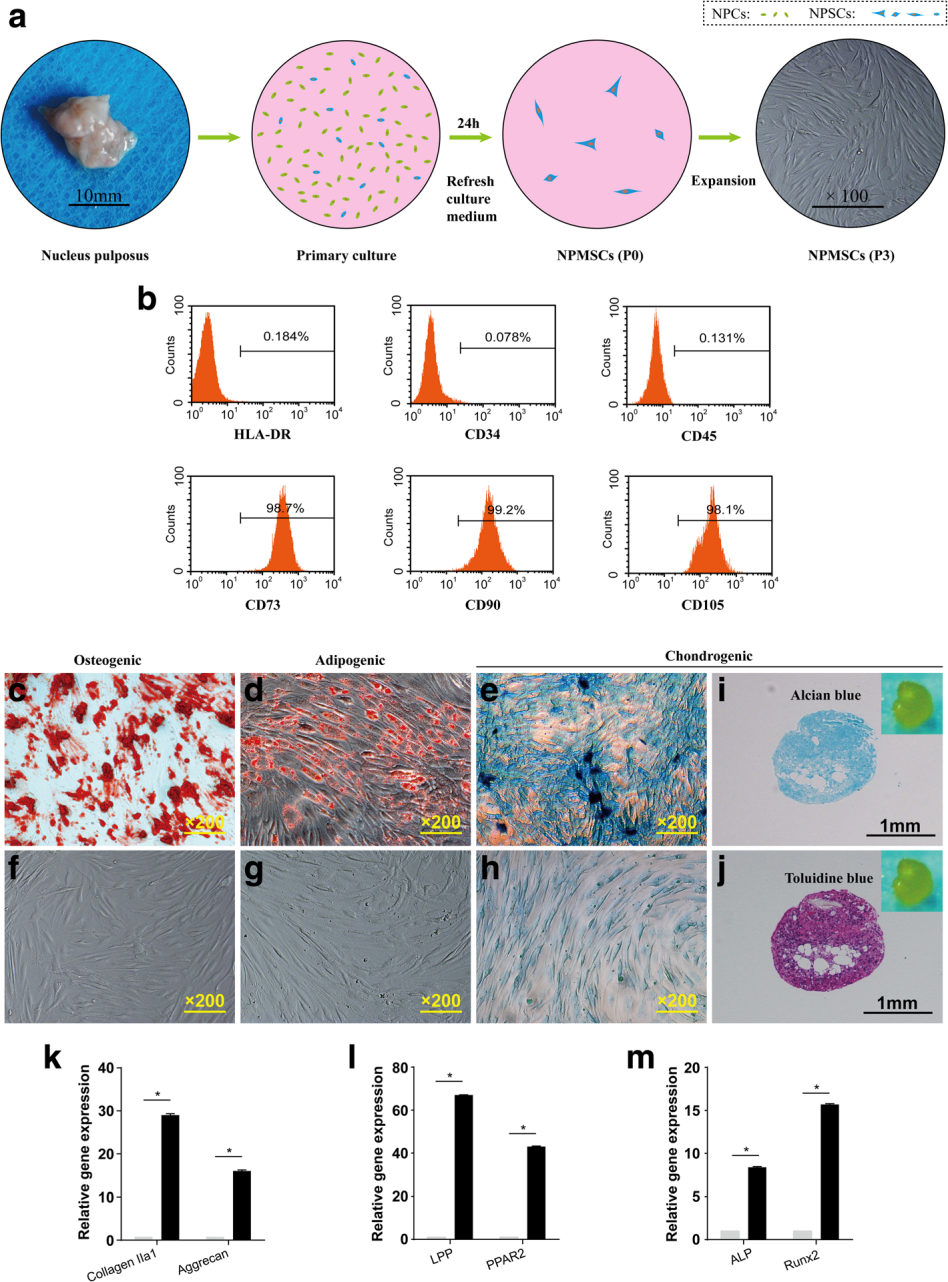
Isolation and identification of human nucleus pulposus mesenchymal stem cells (NPMSCs). **a** Flow diagram of the separation and purification of NPMSCs from human nucleus pulposus (NP) tissue. The harvested NPMSCs at passage 3 displayed a spindle shape in spiral or parallel arrangement. **b** Identification of the stem cell surface molecular profile indicated that the harvested cells were negative for HLA-DR, CD34, and CD45 expression, but positive for CD73, CD90, and CD105 expression. Osteogenic differentiation of NPMSCs (**c**) and control cells (**f**) stained with alizarin red after 3 weeks. Adipogenic differentiation of NPMSCs (**d**) and control cells (**g**) stained with oil red O after 3 weeks. Chondrogenic differentiation of NPMSCs (**e**) and control cells (**h**) stained with Alcian blue after 3 weeks. Identification of chondrogenic microspheres by alcian blue (**i**) and toluidine blue (**j**) staining, respectively. Higher mRNA expression of collagen IIα1 and aggrecan was observed in NPMSCs after a 4-week induction (**k**). Quantitative mRNA analysis of the expression of markers of the three lineages in both induced and control cells showed higher mRNA expression levels of all osteogenic (**k**), adipogenic (**l**), and chondrogenic (**m**) differentiation-related gene expression

After 24 h, the suspended cells and medium were removed, and the adherent cells were cultured and expanded by completely replacing the medium every 2–3 days. As the cells reached 70–80% confluency, the primary cells were harvested and passaged. Passage 1 (P1) NPMSCs were harvested with 0.25% trypsin-ethylenediaminetetraacetic acid (EDTA; Sigma) for 1 min and subcultured at a ratio of 1:3. After the cells were gradually passaged, P3 cells were harvested for identification and cryopreserved for experiments (Fig. 2a).

### Cell viability assay for NC-rich NP explant model

To assess NC viability in the NC-rich NP explant model after culturing for 7 days, NC-rich NP explants were dyed with fluorogenic ester calcein-AM (CAM; Dojindo) to detect live cells, and with propidium iodide (PI; Sigma-Aldrich) to detect dead cells. The tissues were incubated with 2 mM CAM and 4.5 mM PI for 30 min at 37 °C in the dark and gently washed with PBS three times. A fluorescence microscope (CFM-300; Nikon) was used for image acquisition.

### Senescence-associated β-galactosidase (SA-β-gal) staining

After 7 days of incubation, NPMSCs were analyzed using a Senescence β-Galactosidase Staining Kit (Beyotime Institute of Biotechnology). Briefly, cells were washed with PBS, fixed in the SA-β-gal fixative solution for 15 min at room temperature, rinsed three times with PBS, and then incubated in SA-β-gal working solution (Reagents A, B, C, and X-Gal) overnight at 37 °C under atmospheric conditions. Quantification was performed by counting the number of SA-β-gal-positive cells and the total number of cells from three randomly selected areas for each sample.

### Flow cytometry

Cells were harvested and the cell pellet was resuspended in PBS to 10^6^ cells/100 μl. The cell suspension was incubated with the following antibodies: HLA-DR-APC, CD34-FITC, CD45-PE, CD73-PE, CD90-FITC, and CD105-FITC (eBioscience). An isotype control antibody (eBioscience) was used for each. Approximately 5 μl of each antibody (1:10 dilution, according to the antibody datasheets) per tube was added, and this was incubated for 15 min in the dark at 2–8 °C. Cells were completely washed with PBS and resuspended in 1% (w/v) paraformaldehyde (Sangon Biotech, China). Samples were subjected to flow cytometry (BD Biosciences, USA) and the data were analyzed by FlowJo software (FlowJo LLC, Ashland, OR, USA).

### Trilineage differentiation assay

We tested the multidifferentiation potential of NPMSCs toward osteogenic, adipogenic, and chondrogenic lineages in vitro as previously described [1]. Briefly, osteogenic differentiation of MSCs was induced with 10% (v/v) FBS, 1 mM β-glycerol phosphate, 10^−8^ M dexamethasone, and 50 μg/ml ascorbic acid (Cyagen, China). Adipogenic differentiation was induced with 10% (v/v) FBS, 500 μM 1-methyl-3-isobutylxanthine, 10^−9^ M dexamethasone, and 60 μM indomethacin (Cyagen). Chondrogenic differentiation was induced in cell and micromass culture in the presence of 10 ng/ml transforming growth factor (TGF)-β3, 50 μg/ml ascorbic acid, 1% (v/v) insulin-transferrin-selenium (ITS) solution, 10^−7^ M dexamethasone, and 100 μM sodium pyruvate (Cyagen).

Induction was stopped after 3 weeks and alizarin red staining, oil red O staining, and toluidine blue and alcian blue staining were performed to assess osteogenic, adipogenic, and chondrogenic differentiation, respectively.

2.7.1 Alizarin red staining

To identify mineral deposits by alizarin red staining, cells in culture medium were fixed with 70% ethanol for 10 min and stained with 0.5% alizarin red (pH 4.1) for 10 min.

2.7.2 Oil red O staining

To localize lipid droplets, cell layers were fixed with 4% paraformaldehyde for 30 min and incubated in oil red O solution for 15 min. Finally, the cultures were extensively washed with water to remove excess stain and then microscopically observed.

2.7.3 Alcian blue staining or toluidine blue staining

To detect chondrogenic differentiation, cells were rinsed three times with PBS, fixed with 4% paraformaldehyde for 15 min at room temperature, washed with PBS, and stained with 1% alcian blue for 15 min. Finally, the cultures were extensively washed with water to remove excess stain and then microscopically observed. Moreover, to visualize the deposition of sulfated glycosaminoglycans (GAGs), slides were incubated with 1% alcian blue or 1% toluidine blue in 0.1 M HCl overnight. Finally, cultures were washed extensively with distilled water and photographed.

### Cell proliferation assay

To measure cell proliferation, a Cell Counting Kit-8 (CCK-8; Dojindo Laboratories, Japan) was used as previously described [3]. Briefly, NPMSCs were seeded in 24-well plates (1 × 10^4^ cells/well) and different groups were incubated for 1, 3, 5, and 7 days. After removing the culture medium and NC-rich NP explant, 10 μl CCK-8 solution was added to 100 μl fresh medium and the mixture was incubated at 37 °C for 1 h. Finally, the samples were added to 96-well plates for final measurements. Absorbance was measured at 450 nm using a microplate absorbance reader (Bio-Rad, USA). A blank 96-well plate was used for the zero setting. In addition, cell numbers in 24-well plates (1 × 10^4^ cells/well) were also calculated after culturing for 1, 3, 5, and 7 days. All experiments were performed three times for every sample.

### Immunofluorescence microscopy

First-generation NPMSCs were plated in flat-bottomed 24-well plates (1 × 10^4^/well) and fixed with 4% paraformaldehyde, permeabilized with 0.2% triton X-100 in PBS (PBS-T) for 10 min, blocked with PBS containing 5% FBS, and incubated with antibodies against collagen II and carbonic anhydrase 12 (CA12) (1:100, Abcam, UK) at 4 °C overnight. As a negative control, cells were incubated with isotype IgG control antibodies under similar conditions. After the cells were washed, they were incubated with anti-rabbit secondary antibody (Jackson, USA) at a dilution of 1:100 for 1 h at room temperature. Following this, cell nuclei were stained with DAPI solution (1:1000; Invitrogen) for 5 min at room temperature. The samples were examined and photographed using a fluorescence microscope (FV-1000; Olympus). For quantitative examination, the immunostaining results for the animal specimens were analyzed using Image-Pro Plus software (Version 5.1, Media Cybernetics, Inc. USA).

### Western blot assay of type II collagen and CA12

Protein extracts from NPMSCs were prepared in 2 × SDS lysis buffer containing phosphatase and proteinase inhibitors. Protein extracts were subjected to 6% or 12% SDS-PAGE. Protein was separated and then transferred to a polyvinylidene fluoride membranes by electroblotting. The membrane was rinsed in water and blocked with freshly prepared PBS containing nonfat dry milk (5%) for 60 min at room temperature with constant agitation. The membrane was then incubated with human type II collagen (1:5000, Abcam, UK) and CA12 (1:1000, Abcam, UK) antibodies overnight at 4 °C with agitation. After washing the membrane three times with PBS-T, a secondary goat anti-mouse horseradish peroxidase (HRP)-conjugated antibody was added and incubated at room temperature for 2 h. Following another three washes with PBS-T, protein bands were visualized using the LiCoR Odyssey imager (LI-COR Biosciences, Lincoln, NE, USA), and semiquantification was performed using the LiCoR Odyssey imager software.

### Biochemical evaluation of GAG/DNA

The glycosaminoglycan (GAG) content was determined by performing a dimethylmethylene blue (DMMB) assay [29]. Briefly, after the NPMSCs were washed with PBS three times to eliminate the influence of NP explant tissue or culture medium, NPMSCs were incubated in solution containing 1 mg/ml papain in 100 mM sodium phosphate (pH 6.5), 5 mM l-cysteine, and 5 mM EDTA overnight at 60 °C. The GAG content in NPMSCs was determined by the reaction with DMMB, and optical density was measured at 525 nm using a microplate reader [30–32]. GAG concentrations were calculated using a standard curve obtained from chondroitin sulfate standards (Sigma). The DNA content of the samples was measured with bisbenzimidol fluorescent dye (Hoechst 33,258; Sigma). The standard curve was obtained with known concentrations of calf thymus DNA. The amount of DNA in each sample was calculated based on the standard curve.

### Real-time polymerase chain reaction (RT-PCR) analysis

The expression of genes encoding markers of senescence (p16, p21, and p53), senescence-associated proinflammatory cytokines (interleukin (IL)-6), extracellular proteases (MMP13, ADAMTS-5), a notochordal cell marker (brachyury), NPC markers (FOXF1, Pax-1, CA12, and HIF-1α), ECM molecules (collagen Iα1, collagen IIα1, Sox-9, and aggrecan), and markers for the three lineages (LPL, PPAR2, ALP, Runx2) was analyzed by RT-PCR. The primers used in this study are shown in Table 1 (Sangon Biotech Corporation). Briefly, after NPMSCs were incubated under different conditions for 7 days, 1 μg RNA, extracted using TriPure Isolation Reagent (Roche, Switzerland), was reverse-transcribed into cDNA using a First Strand cDNA Synthesis Kit (Roche) as per the manufacturer’s instructions. Then, a reaction mixture containing cDNA, SYBR Green Mix (Toyobo, Japan), and primers (Table 1) was subjected to RT-PCR (CFX96 Real-Time System, Bio-Rad) according to the manufacturer’s recommended conditions. β-actin was used as an internal reference, and relative gene expression was determined using the 2^–△△Ct^ method.

**Table 1:** Primers for target genes

Gene	Forward sequence	Reverse sequence
Collagen Iα1	5′-CCTGGAAAGAATGGAGATGATG-3′	5′-ATCCAAACCACTGAAACCTCTG-3′
Collagen IIα1	5′-GGTAAGTGGGGCAAGACTGTTA-3′	5′-TGTTGTTTCTGGGTTCAGGTTT-3′
Sox-9	5′-GCCTCTACTCCACCTTCACCTA-3′	5′-GCTGTGTGTAGACAAGTTGTT-3′
Aggrecan	5′-GTCAGATACCCCATCCACACTC-3′	5′-CATAAAAGACCTCACCCTCCAT-3′
LPL	5’-TCCGCGTGATTGCAGAGAGAG-3′	5’-TGCTGCTTCTTTTGGC TCTGACT-3′
PPAR-2	5’-CGAGGGCGATCTTGACAGGAA − 3′	5’-CAGGGGGGTGATGT GTTTGAAC- 3′
RUNX-2	5’-ACGACAACCGCACCATGGT-3’	5’-CTGTAATCTGACTCT GTCCT-3’
ALP	5’-TGGAGCTTCAGA AGCTCAACACCA-3’	5’-ATCTCGTTGTCTGAG TACCAGTCC-3’
p16	5′-ACCAGAGGCAGTAACCATGC-3′	5′-TGATCTAAGTTTCCCGAGGTTT-3′
p21	5′-TTAGCAGCGGAACAAGGAGT-3′	5′-CGTTAGTGCCAGGAAAGACA-3′
p53	5′-TAGTGTGGTGGTGCCCTATG-3′	5′-CCAGTGTGATGATGGTGAGG-3′
IL-6	5′-ATGCCTGACCTCAACTCCACT-3′	5′-GCCACCCAGCTGCAAGATTTC-3′
ADAMTS-5	5′-GGACCTACCACGAAAGCAGATC-3′	5′-GCCGGGACACACGGAGTAC-3′
MMP-13	5′-TGGAAGGATGCCTTTTTTTCTC-3′	5′-CACCCTCCCCAAGTATCAATAGG-3′
Brachyury	5′-AGACAGCCAGCAATCTG-3′	5′-TGGAGGGAAGTGAGAGG-3′
KRT18	5′-GGACAGCTCTGACTCCAGGT-3′	5′-AGCTTGGAGAACAGCCTGAG-3′
HIF-1α	5′-GAAGTGTACCCTAACTAGCCGAGGA-3′	5′-TGAATGTGGCCTGTGCAGTG-3′
CA12	5′-CTGTGGGTCCAGCTTTGGAA-3′	5′-TGCCATGCAGCAGAGTTAGGAG-3′
FOX1	5′-CACACAGGAATTCTGCTGAGGT-3′	5′-TGATTGGTCTCACATGTTTGCT-3′
PAX1	5′-AACTTGGCTTAAATCTCTGCTCCAC-3′	5′-GCATTGAAGGCTACATTTCACAGAC-3′
β-actin	5′-GTGGGGCGCCCCAGGCACCA-3′	5′-CTTCCTTAATGTCAC GCACGATTTC-3′

### Statistical analysis

The mean values obtained were compared by analysis of variance (ANOVA) with SPSS13.0 software (SPSS). Data are presented as mean ± standard deviation values. A Student’s *t* test or one-way ANOVA was used for comparisons between groups; differences were considered statistically significant at *P* values < 0.05.

## Results

### Identification of human NPMSCs

Cells isolated from human degenerated IVDs displayed fibroblast-like or spindle-shaped morphology in monolayer culture, indicating plastic adhesion ability (Fig. 2a). Based on immune phenotypic assays, cells were positive for CD73, CD90, and CD105 expression (> 95%, Fig. 2b) and negative for CD34, CD45, and HLA-DR (< 2%, Fig. 2b). In multilineage differentiation tests, all cells were capable of osteogenic, chondrogenic, and adipogenic differentiation in the respective types of differentiation induction media after 3 weeks (Fig. 2c–j). Moreover, the expression level of osteogenic, chondrogenic, and adipogenic differentiation markers were significantly increased in all induction groups compared with those in the control groups (Fig. 2k–m). In summary, the obtained cells met the ISCT criteria for NPMSCs, including adhesive characteristics, SC phenotypic expression, and multilineage differentiation potential.

### NC morphology, phenotype, and viability in NC-rich NP explant model

**Fig. 3:**
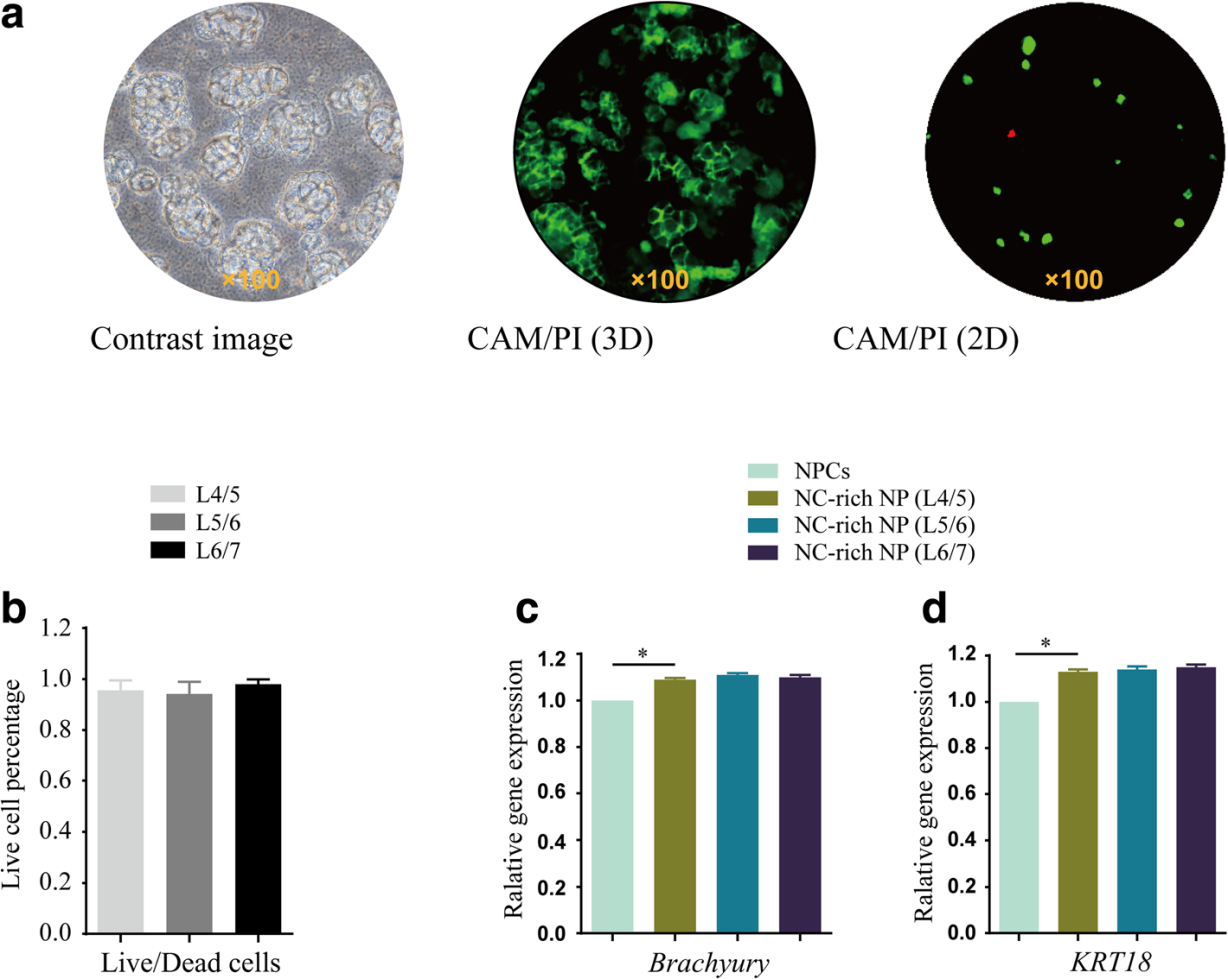
Notochordal cell (NC) viability and markers in NC-rich nucleus pulposus (NP) explant model after 7 days of culture. The viability of NCs in NC-rich NP explants derived from L4/5, L5/6, or L6/7 IVDs is shown, based on similar live cell percentages (**a,b**). For real-time PCR, brachyury (**c**) and keratin 18 (KRT18) (**d**) were tested in the three segments of NC-rich NP explants, which were found to be similarly expressed but significantly upregulated compared with expression in NCs. All data are expressed as mean ± standard deviation; *n* = 3. CAM calcein-AM, PI propidium iodide

After 7 days of culture, the NCs cultured in ECM were found to be agminated in several small cluster structures (Fig. 3a). Cell morphologies were round or polygonal with different sized vacuoles in the cytoplasm (Fig. 3a). For cell viability, CAM/PI staining was used, and green staining indicated viability whereas dead cells were stained red (Fig. 3a). NCs from L4/5, L5/6, and L6/7 segments exhibited similar live cell percentages, with 94 ± 5% viable cells in the L4/5 segment, 92 ± 7% in the L5/6, and 98 ± 2% in the L6/7 (Fig. 3b). In addition, the expression levels of the NC markers brachyury and keratin 18 (KRT18) were also similar, with no significant differences among the three groups after 7 days of culture, whereas the expression of both genes was significantly higher than that in NCs (Fig. 3c, d).

### NC-rich NP explants decrease cell senescence in TNF-α-treated NPMSCs

**Fig. 4:**
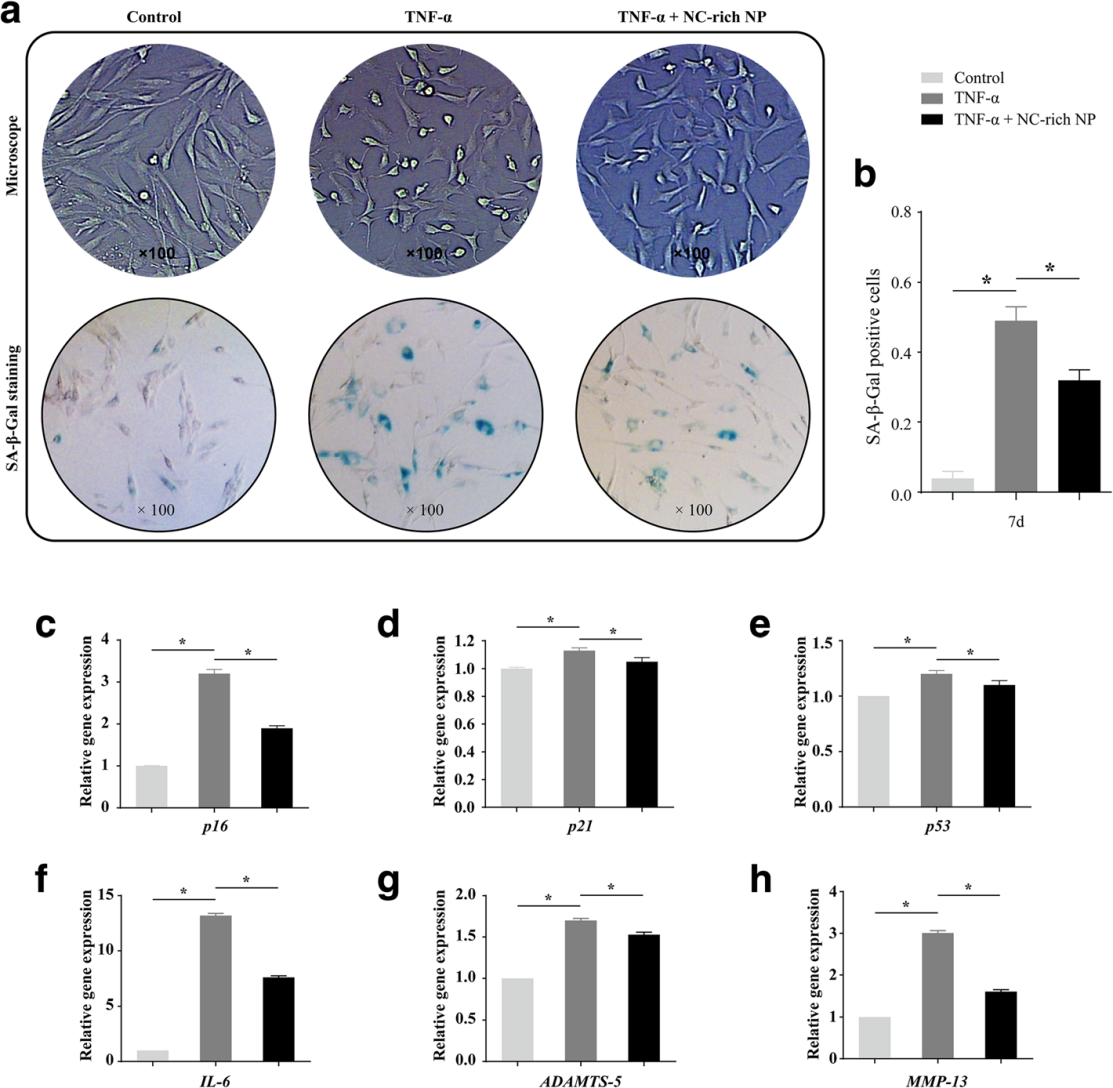
Coculturing tumor necrosis factor (TNF)-α-treated nucleus pulposus mesenchymal stem cells (NPMSCs) with notochordal cell (NC)-rich nucleus pulposus (NP) explants attenuates cell senescence. Morphological changes in NPMSCs with different test compounds, as detected by inverted microscopy, scanning electron microscopy, and senescence-associated β-galactosidase (SA-β-gal) staining after 7 days of culture (**a**). Positive staining for SA-β-gal was compared in different groups (**b**). Meanwhile, real-time PCR analysis of p16 (**c**), p21 (**d**), and p53 (**e**) mRNA expression was performed. Furthermore, the expression of senescence-associated proinflammatory cytokines interleukin (IL)-6 (**f**) and extracellular proteases MMP-13 (**g**) and ADAMTS-5 (**h**) was tested. Data are expressed as mean ± SD values (*n* = 4). *p < 0.05

In terms of cell morphology, NPMSCs in the control group showed compact parallel or vortex populations, whereas the TNF-α-treated cells exhibited disorderly distribution after 7 days of culture (Fig. 4a). In addition, NPMSCs in the control group had elongated spindle shapes, whereas TNF-α-treated cells had many slender processes (Fig. 4a). However, this senescent cell morphology was somewhat attenuated upon coculture with NC-rich NP explants (Fig. 4a). Furthermore, cell senescence was analyzed by SA-β-gal staining (Fig. 4a). An increased number of SA-β-gal-positive NPMSCs was observed with TNF-α treatment compared with that in the control group after 7 days (*P* < 0.05; Fig. 4b). However, when TNF-α-treated cells were cocultured with NC-rich NP explants, a reduction in SA-β-gal-positive cells was observed, and this difference was significant (*P* < 0.05; Fig. 4b). For cell senescence-related gene analysis, TNF-α significantly upregulated the expression of p16, p21, and p53 compared with that in the control group. However, NC-rich NP explants decreased the expression of these markers after TNF-α treatment (*P* < 0.05; Fig. 4c–e). Finally, the expression of senescence-associated proinflammatory cytokines (IL-6) and extracellular proteases (MMP-13, ADAMTS-5) was tested, and TNF-α was found to increase the expression of all three genes compared with that in the control group; in contrast coculturing with NC-rich NP explants led to a decline in these markers (*P* < 0.05; Fig. 4f–h). Taken together, these results indicate that NC-rich NP explants can attenuate TNF-α-induced NPMSC senescence.

### NC-rich NP explants promote the proliferation of TNF-α-treated NPMSCs

**Fig. 5:**
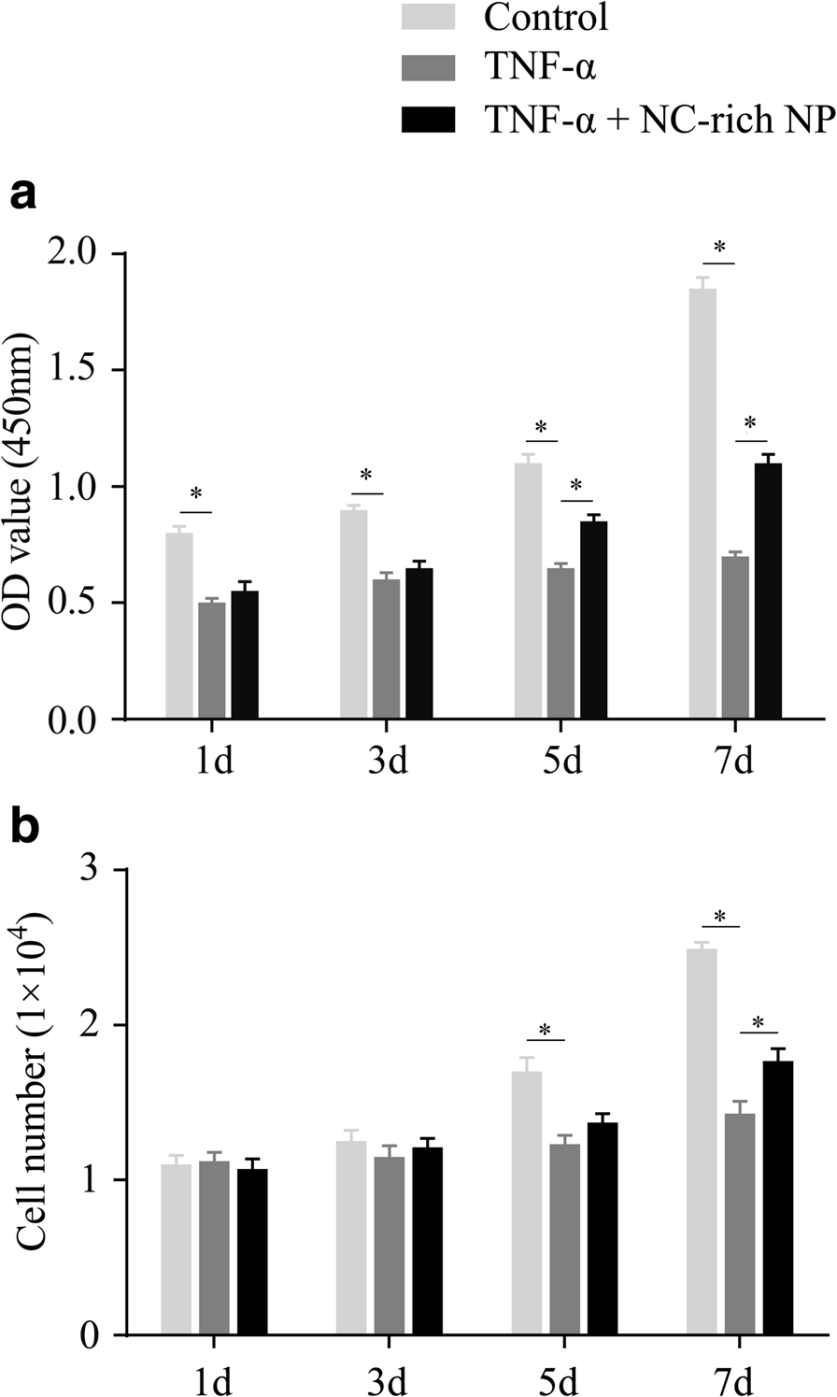
Coculturing tumor necrosis factor (TNF)-α-treated nucleus pulposus mesenchymal stem cells (NPMSCs) with notochordal cell (NC)-rich nucleus pulposus (NP) explants significantly promotes proliferation. After NPMSCs were treated with different test compounds for 7 days, cell proliferation assays (resulting in optical density (OD) values) were performed. Proliferation in TNF-α-treated NPMSCs was significant decreased on days 1, 3, 5, and 7, whereas coculturing with NC-rich NP explants attenuated the inhibitory effects of TNF-α on NPMSC proliferation, with significant differences on days 5 and 7 (**a**). In addition, NPMSC numbers were similar but were associated with a significant decline on days 5 and 7; however, coculturing with NC-rich NP explants attenuated this trend on day 7 (**b**). Data are expressed as mean ± SD values (*n* = 4). *P < 0.05

Cell growth curves were used to evaluate the proliferative ability of NPMSCs after performing CCK-8 assays. The optical density (OD) value of TNF-α-treated NPMSCs was significantly lower than that of the control group on days 1, 3, 5, and 7 (*P* < 0.05; Fig. 5). However, coculture with NC-rich NP explants increased the proliferation of TNF-α-treated NPMSCs, as indicated by markedly higher OD values on days 5 and 7 (*P* < 0.05; Fig. 5a). Additionally, significantly lower numbers of NPMSCs were detected in the coculture group compared with that in the TNF-α-treated group on days 5 and 7 (both *P* < 0.05; Fig. 5b), while in both TNF-α-treated NPMSCs, a higher cell number was observed in the NC-rich NP coculture group, with a significant difference at day 7 (*P* < 0.05; Fig. 5b). These results suggested that NC-rich NP can attenuate the inhibitory effects of TNF-α on NPMSC proliferation.

### NC-rich NP explants enhance the expression of NP cell markers in TNF-α-treated NPMSCs

**Fig. 6:**
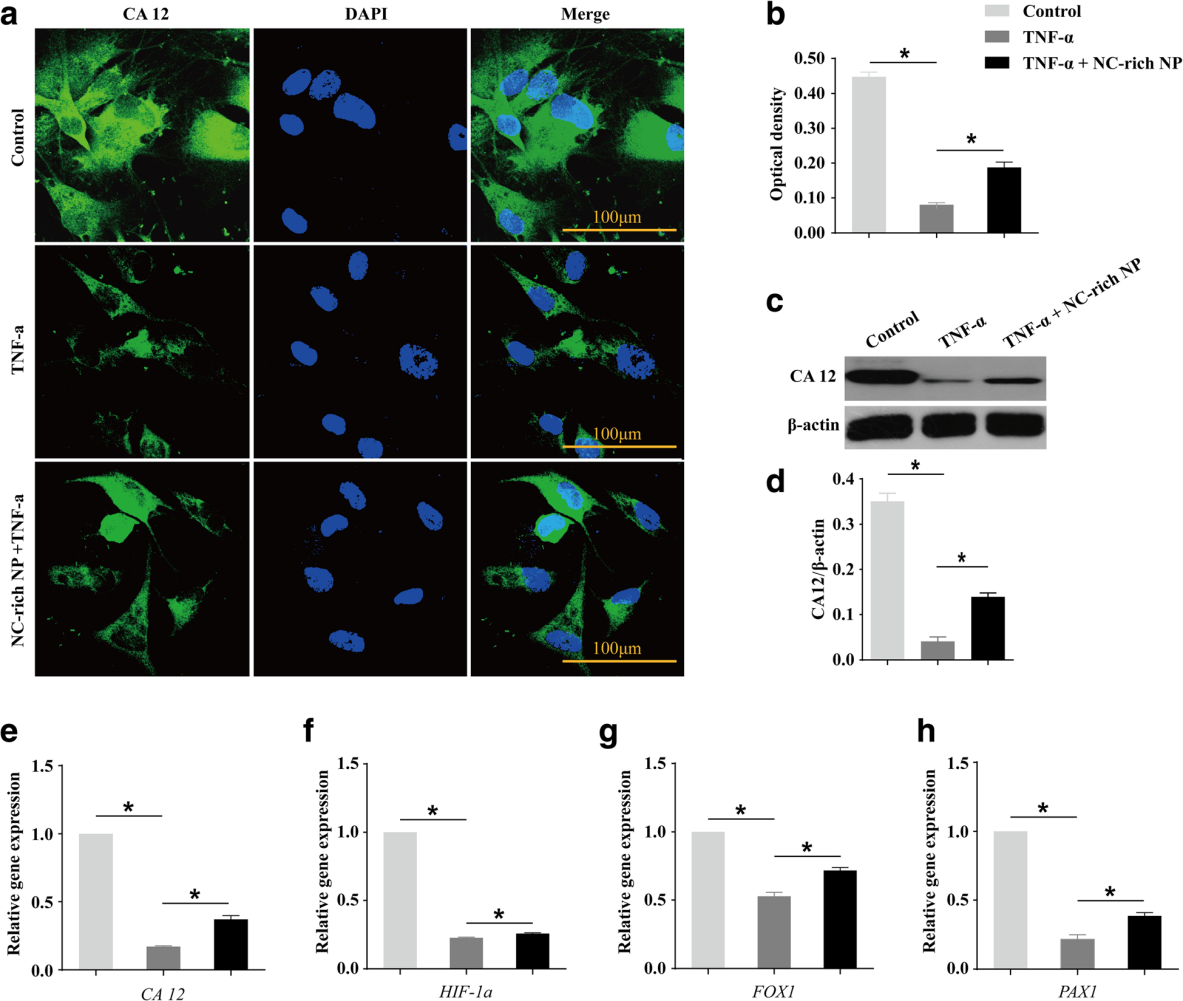
Coculturing tumor necrosis factor (TNF)-α-treated nucleus pulposus mesenchymal stem cells (NPMSCs) with notochordal cell (NC)-rich nucleus pulposus (NP) explants enhances the expression of NP cell markers. Representative image of immunofluorescence staining (**a**) and quantification analysis of carbonic anhydrase 12 (CA12) protein expression in the three groups after 7 days of culture (**b**). Western blot analysis (**c**) and densitometric quantification (**d**) of CA12 proteins with different treatments. The gene expression levels of CA12, FOXF1, PAX1, and HIF-1α in NPMSCs were compared among different groups after 7 days of culture (**e**–**h**). All data are expressed as mean ± SD values (*n* = 4). *P < 0.05

CA12 is known as a specific marker of NP cells; here, we examined the expression of this marker in TNF-α-treated NPMSCs with or without coculture with NC-rich NP explants. As shown in Fig. 6a, b, the expression of CA12 was downregulated in TNF-α-treated cells compared with that in the control condition; however, it was upregulated upon coculture with NC-rich NP explants (*P* < 0.05). Western blot analysis also showed that the level of CA12 was decreased in the TNF-α-treated condition but was increased with NC-rich NP explant coculture (*P* < 0.05; Fig. 6c, d). A similar trend was found based on RT-PCR analysis of the NP cell markers CA12, Forkhead box F1 (FOXF1), and paired box 1 (PAX1) (*P* < 0.05; Fig. 6e, g, h). Furthermore, hypoxia inducible factor (HIF)-1α is continuously expressed in NP cells and plays a pivotal role in NP biology by precisely regulating essential cellular functions such as differentiation and survival [33]. In this study, NC-rich NP explants significantly inhibited TNF-α-mediated downregulation of HIF-1α levels compared with the expression in untreated groups, indicating that the application of NC-rich NP explants can significantly promote differentiation into nucleus pulposus-like cells (*P* < 0.05; Fig. 6f).

### NC-rich NP explants increase ECM-related gene expression and GAG/DNA in TNF-α-treated NPMSCs

**Fig. 7:**
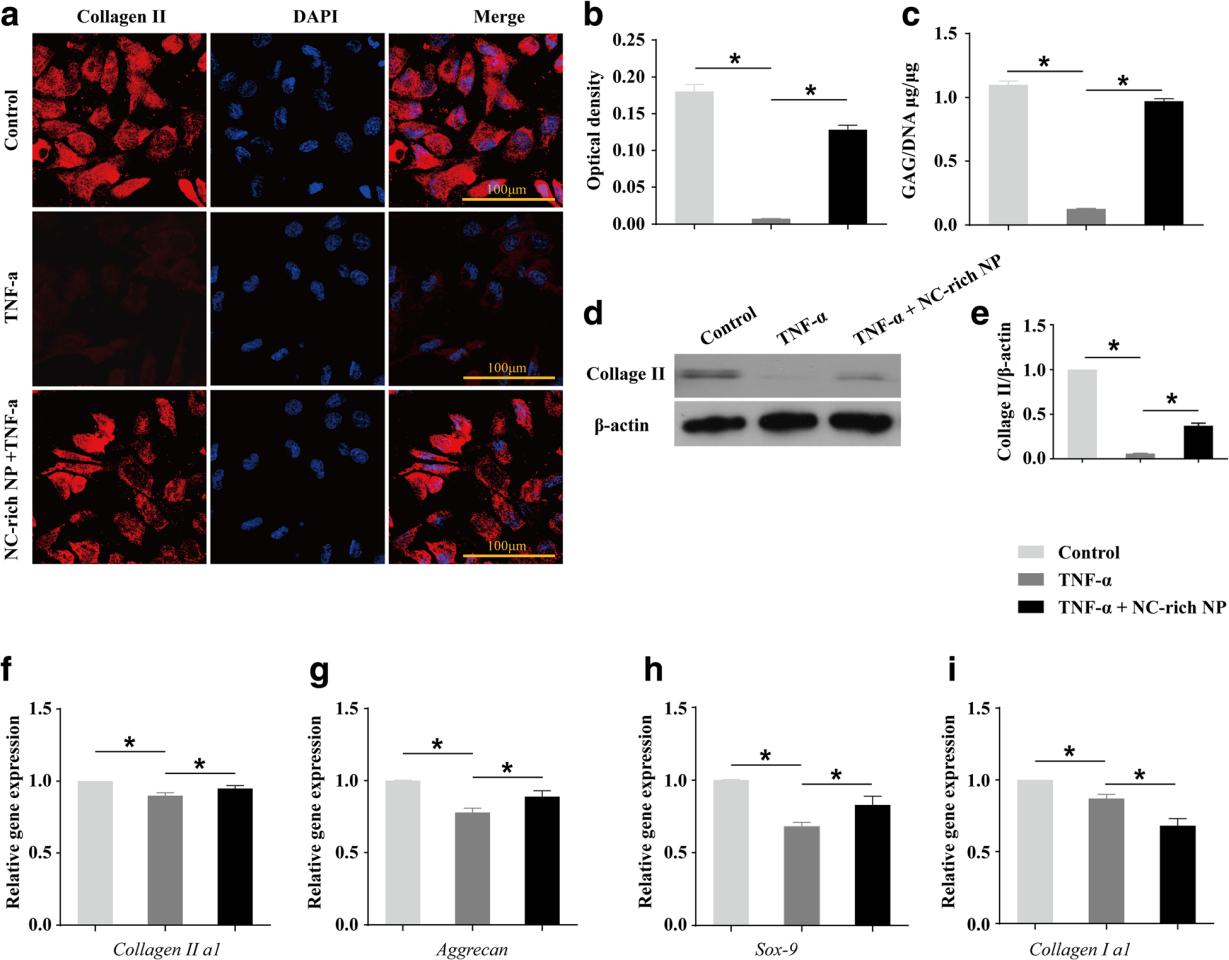
Coculturing tumor necrosis factor (TNF)-α-treated nucleus pulposus mesenchymal stem cells (NPMSCs) with notochordal cell (NC)-rich nucleus pulposus (NP) explants increases the expression of matrix macromolecules. Representative image of immunofluorescence staining (**a**) and quantification analysis of collagen II protein expression in the three groups after 7 days of culture (**b**). Glycosaminoglycan (GAG)/DNA ratio in NPMSCs treated with different test compounds for 7 days (**c**). Western blot analysis (**d**) and densitometric quantification (**e**) of collagen II proteins in three groups. The mRNA expression levels of aggrecan, Sox-9, Col1α1, and Col2α1 in NPMSCs were compared within different groups after 7 days of culture (**f**–**i**). All data are expressed as mean ± SD values (*n* = 4). *P < 0.05

To evaluate the effect of NC-rich NP on the biological synthesis of ECM, immunofluorescence for collagen II and the GAG/DNA ratio was analyzed in NPMSCs. The collagen II optical density and the GAG/DNA ratio significantly decreased in the TNF-α-treated group (Fig. 7a–c). However, this was found to be markedly inhibited in the coculture group (Fig. 7a–c). Western blot analysis also displayed the same trend, where collagen II was decreased in TNF-α-treated medium and increased in the coculture group (Fig. 7d, e). Finally, we also investigated matrix synthesis by evaluating the expression of genes encoding collagen type Iα1, collagen type IIα1, aggrecan, and Sox-9 (Fig. 7f–i). Although matrix molecule (aggrecan, Sox-9, and collagen IIα1) expression decreased in TNF-α-treated NPMSCs, NC-rich NP significantly abolished this inhibitory effect (Fig. 7f–h). Conversely, collagen type Iα1 expression was shown to decrease further in the coculture group compared with that in the TNF-α-treated group (Fig. 7i). Collectively, these results indicate that NC-rich NP can promote matrix synthesis in TNF-α-treated NPMSCs.

## Discussion

NCs and NCCM have been reported to possess regenerative potential for the treatment of IDD [34–36]. However, whether this ability can also be effective for endogenous NPMSCs remains unclear. In addition, although NCs or the application of NCCM has been associated with numerous effects during the repair of IDD, the degeneration of the native NP tissue environment and the loss of some therapeutic factors during production and cryopreservation have limited further study [25]. Therefore, a modified NC-rich NP explant culture model involving partial digestion of NP tissue was used in our previous study [28]. This improved NC-rich NP culture model demonstrated promising repair potential for human NPCs by prolonging cell viability and maintaining native environments. Based on that, the effect of coculturing NPMSCs with NC-rich NP explants with TNF-α treatment was investigated in the present study. These inflammatory conditions might indicate a more compelling effect and accordingly provide additional incentive to produce sufficient ECM and resident cells during IDD.

To date, although a number of studies have shown the activating effects of NCs for the treatment of IDD, the regeneration potential was mild or invalid due to the disruption of the physiological organization of NCs [4, 37, 38]. In this study, cell viability and the expression of NC phenotype-related genes after NC-rich NP explant were tested in rabbit L4/5, L5/6, and L6/7 IVDs; the results demonstrated no significant differences, indicating that the NC-rich NP explant model was comparable among the three segments used in this study and that these could be used randomly. Hence, a series of experiments was performed in the current study using the NC-rich NP–NPMSC coculture system. We found that NC-rich NP explants could stimulate the anabolism of degenerative NPMSCs by enhancing cell proliferation, decreasing cellular senescence, and promoting GAG, collagen II, and CA12 production, together with upregulating the expression of ECM-related genes (collagen I, collagen II, SOX9, and ACAN) and enhancing the phenotype of NPCs (HIF-1α, FOXF1, PAX1, and CA12 expression). Collectively, our results demonstrate that NC-rich NP explants can potentially promote a nondegenerated NP phenotype. Our results are in accordance with some similar studies on the regenerative potential of NCs; specifically, these cells were thought to produce nutritional components or secrete growth factors [23, 39]. Importantly, factors secreted by NCs isolated from different species can have cross-species regenerative effects on human degenerative cells [22].

Inflammation is the main pathological process associated with disc degeneration, and TNF-α, as a typical inflammatory cytokine, plays a crucial role in inflammatory cytokine-induced disc degeneration [40]. In this study, TNF-α was added to the culture medium to induce a degenerative environment similar to IDD, thereby simplifying the complex inflammatory milieu. This differed considerably from the results of numerous previous studies regarding physiological conditions, further suggesting the beneficial potential of NC-rich NP explants on NPMSCs for the treatment of IDD. In the present study, TNF-α successfully induced premature senescence and detrimentally affected properties such as cell morphology, cell proliferation, cell senescence, and the expression of matrix macromolecules in NPMSCs. Subsequently, NC-rich NP explants were shown to exhibit an anti-inflammatory effect during IDD by inhibiting these adverse outcomes, similar to that observed for NPCs used in our previous studies [39]. Therefore, the therapeutic function of NC-rich NP explants represents a promising strategy for preventing and treating IDD.

NPMSCs are considered to play a crucial role in the maintenance of normal NP tissue homeostasis [41]. However, the gradual increase in NPMSC senescence during IDD has a detrimental effect, and inhibiting this senescence of NPMSCs is thus considered an important strategy for the treatment of IDD [42–44]. Our results suggest that NC-rich NP explants can attenuate the premature senescence of NPMSCs in an inflammatory microenvironment. The telomere-based p53-p21-pRb and the stress-based p16-pRb pathways are predominant pathways in IDD [19]. Our results suggest that both pathways are involved in the effect of TNF-α on NPMSCs in vitro, and the extrinsic factor p16 was shown to play a crucial role as demonstrated by its marked upregulation. Moreover, this study also demonstrates that coculture with NC-rich NP can result in the downregulation of senescence-associated proinflammatory cytokines (IL-6) and extracellular proteases (MMP-13, ADAMTS-5), which further strengthens our conclusion.

During the progression of IDD, NPMSC numbers decrease quickly [15, 45, 46]. Therefore, regeneration therapy including promoting cell proliferation is a promising and feasible option. In accordance with the positive effects of NCCM and NCs [24], NPMSC proliferation decreased in the TNF-α-treated group, whereas proliferation was restored upon NC-rich NP–NPMSC coculture. Furthermore, analysis indicated that the increase in cell number was due to cell proliferation rather than the inhibition of cell death. These data are of great importance for developing potential treatments aimed at inhibiting the reduction in cell number that is observed during IDD.

To evaluate phenotypic alterations in NPMSCs, we also examined the expression of NP cell markers following different treatments. It has been shown that TNF-α can downregulate the expression NP markers, leading to altered expression of NP-associated markers such as CA12, FOXF1, PAX1, and HIF-1α. However, the application of NC-rich NP explants had a significant protective effect on the expression of HIF-1α, FOXF1, PAX1, and CA12. It has been noted that FOXF1, PAX1, and CA12 are highly expressed in NP cells and that the expression of HIF-1α is closely related to cell function in low-oxygen environments. Our results suggest that NC-rich NP explants can promote NPMSC differentiation into the NP cell phenotype.

Apart from the above, the decline in ECM is also a key issue. The balance between ECM anabolism and catabolism by disc cells is disturbed by proinflammatory cytokines during IDD [47, 48]. Hence, inhibition of these inflammatory cytokine-mediated pathological processes might promote NPMSC differentiation and result in increased deposition of ECM. Our study proved that NC-rich NP explants can increase matrix synthesis by promoting gene expression of aggrecan and collagen IIα1 and the protein synthesis of collagen II and GAG. In addition, the expression of Sox-9, indicative of a healthy NP phenotype, was higher in the coculture group than in the TNF-α-treated group. This finding further indicates that NC-rich NP explants have the potential to stimulate degenerative NPMSCs to differentiate into NP-like cells. In addition, the reduction in collagen Iα1 expression suggests that the inhibitory effect of NC-rich NP during fibrosis further facilitates NPMSC differentiation. Taken together, these results indicate that NC-rich NP explants can promote matrix synthesis in TNF-α-treated NPMSCs.

This study also has some limitations. Although TNF-α can mimic the inflammatory effects of IDD to some extent, the actual environment associated with this condition is complex, with various factors that are difficult to completely recapitulate. Furthermore, although the conclusion was based on in-vitro evidence, there is no precise animal model to examine the therapeutic effects on disc degeneration in vivo. Although the positive effect of NC-rich NP on degenerated NPMSCs during IDD regeneration was shown, the exact mechanism is still unclear and requires further study. The next step would be to determine this mechanism and to confirm this in vivo using animal models of IDD.

## Conclusions

In conclusion, our study reveals that modified NC-rich NP explants have a positive effect on NPMSCs in an anti-inflammatory microenvironment by increasing cell proliferation, decreasing cellular senescence, and promoting ECM accumulation. Therefore, our results provide novel insights into the therapeutic potential of NC-rich NP for the treatment of IDD and might supply additional motivation to produce sufficient ECM and resident cells for the treatment of IDD.

## Abbreviations

ANOVA: Analysis of variance
CA12: Carbonic anhydrase 12
CAM: Calcein-AM
DMEM: Dulbecco’s modified Eagle’s medium
DMMB: Dimethylmethylene blue
ECM: Extracellular matrix
EDTA: Ethylenediaminetetraacetic acid
FBS: Fetal bovine serum
FOXF1: Forkhead box F1
GAG: Glycosaminoglycan
HIF: Hypoxia inducible factor
IDD: Intervertebral disc degeneration
IL: Interleukin
IVD: Intervertebral disc
KRT18: Keratin 18
MSC: Mesenchymal stem cell
NCCM: Notochordal cell-conditioned medium
NC: Notochordal cell
NP: Nucleus pulposus
NPC: Nucleus pulposus cell
NPMSC: nucleus pulposus mesenchymal stem cell
OD: Optical density
P: Passage
PAX1: Paired box 1
PBS: Phosphate-buffered saline
PI: Propidium iodide
RT-PCR: Real-time polymerase chain reaction
SA-β-gal: Senescence-associated β-galactosidase
SC: Stem cell
TNF: Tumor necrosis factor
